# Phosphodiesterase 5 (PDE-5) inhibitors (sildenafil, tadalafil, and vardenafil) effects on esophageal motility: a systematic review

**DOI:** 10.1186/s12876-023-02787-3

**Published:** 2023-05-22

**Authors:** Arman Shafiee, Razman Arabzadeh Bahri, Mohammad Mobin Teymouri Athar, Fatemeh Afsharrezaei, Mostafa Gholami

**Affiliations:** 1 Department of Psychiatry and Mental Health, Alborz University of Medical Sciences, Karaj, Iran; 2 Student Research Committee, School of Medicine, Alborz University of Medical Sciences, Karaj, Iran; 3 School of Medicine, Tehran University of Medical Sciences, Tehran, Iran; 4 School of Medicine, Shahid Beheshti University of Medical Sciences, Tehran, Iran; 5 School of Medicine, Alborz University of Medical Sciences, Karaj, Iran

**Keywords:** Esophageal motility, Achalasia, Phosphodiesterase, Sildenafil, Tadalafil, Vardenafil

## Abstract

**Background:**

Esophageal motility disorders are a group of disorders associated with dysfunctional swallowing resulting from impaired neuromuscular coordination. Phosphodiesterase 5 (PDE-5) inhibitors induce smooth relaxation and are proposed as a treatment option for esophageal motility disorders such as achalasia.

**Methods:**

This study is conducted based on the Preferred Reporting Items for Systematic Reviews and Meta-Analyses (PRISMA). We systematically searched MEDLINE/ PubMed, Scopus, EMBASE, and Web of Science databases for esophageal outcomes of individuals treated with PDE5 inhibitors. A random effect meta-analysis was conducted.

**Results:**

A total of 14 studies were included. They were conducted in different countries, with Korea and Italy having the highest number of articles. The main drug assessed was sildenafil. PDE-5 inhibitors resulted in a significant reduction in lower esophageal sphincter pressure (SMD − 1.69, 95% CI: -2.39 to -0.99) and the amplitude of contractions (SMD − 2.04, 95% CI: -2.97 to -1.11). Residual pressure was not significantly different between the placebo and sildenafil groups (SMD − 0.24, 95% CI: -1.20 to 0.72). Furthermore, a recent study reported contractile integral, stating that ingestion of sildenafil leads to a significant reduction in distal contractile integral and a significant increase in proximal contractile integral.

**Conclusion:**

PDE-5 inhibitors significantly reduce LES resting pressure and esophageal peristaltic vigor, decreasing esophageal body contractility and contraction reserve. Therefore, using these drugs in patients affected by esophageal motility disorders may potentially improve their condition regarding symptom relief and prevention of further associated complications. Future reports investigating larger sample size is necessary in order to establish definite evidence regarding the efficacy of these drugs.

**Supplementary Information:**

The online version contains supplementary material available at 10.1186/s12876-023-02787-3.

## Background

Esophageal motility disorders are a group of disorders associated with swallowing dysfunction ensuing from the dysregulation of peristaltic motions of the esophagus caused by abnormalities in neuromuscular structures and their function. These disorders include but not limited to achalasia, nutcracker esophagus, and hypertensive lower esophageal sphincter (LES). Symptoms mainly include dysphagia to solid and liquid, and chest pain; other symptoms such as odynophagia, regurgitation, heartburn, and weight loss could manifest in some of the patients [1]. Moreover, complications following aspiration and respiratory problems might occur as a result of malfunctioned swallowing. Therefore, diagnosis and treatment of these diseases are of great importance in order to both palliate the symptoms and prevent further complications. Achalasia is characterized by impaired LES relaxation in response to swallowing and lack of esophageal peristalsis [2]. The primary pathophysiological mechanism behind achalasia is proposed to be the inhibition of nerve function resulting from selective loss of inhibitory ganglions in the myenteric plexus as a result of cell-mediated and antibody-mediated autoimmune attacks targeting myenteric nerves in the esophagus, followed by a decrease in nitric oxide (NO), the primary inhibitory neurotransmitter of the gastrointestinal tract [3]. Evaluation of achalasia includes endoscopy, high-resolution manometry, and barium radiography. Botulinum toxin injection, pneumatic dilation, and Heller myotomy are available treatments which help with symptom relief [4]. Direct activation of guanylyl cyclase by NO results in an increase in intracellular 3’, 5’-cyclic monophosphate (cGMP), leading to initiation of a signaling cascade which consequently relaxes smooth muscles or reduces contractions. Phosphodiesterase (PDE) type 5 terminates actions of cGMP. PDE5 inhibitors such as sildenafil, tadalafil, and vardenafil cause cGMP accumulation by inhibiting PDE5, which prompts smooth muscle relaxation [5]. Sildenafil is a potent PDE5 inhibitor used in the treatment of erectile dysfunction. Moreover, it is found to decrease the LES pressure and inhibit esophageal propulsive force [6]. The accordance of the pathophysiology of esophageal motility disorders with the mechanism of action of PDE5 inhibitors raises the question of whether PDE5 inhibitors such as sildenafil are effective treatment options for esophageal motility disorders such as achalasia. Moreover, there is a need for less invasive treatments for these disorders due to several adverse effects reported after these invasive treatments [7]. Therefore, we set out to address the existing knowledge gap regarding the benefits of the utility of these medications by systematically reviewing the studies evaluating the efficacy of PDE-5 inhibitors in the possible improvement of esophageal motility disorders.

## Methods

This study is reported based on the Preferred Reporting Items for Systematic Reviews and Meta-Analyses (PRISMA) guidelines.

### Search strategy

We have searched MEDLINE/ PubMed, Scopus, EMBASE, and Web of Science (WoS) databases until December 29, 2021, on esophageal outcomes of individuals treated with PDE-5 inhibitors. On July 14, 2022, a second search was conducted to ensure no additional studies were missed. The following keywords were searched to retrieve relevant studies: (“esophag*“[Title/Abstract] OR ”esophageal“[Title/Abstract] OR ”lower esophageal sphincter“[Title/Abstract] OR ”LES“[Title/Abstract] OR “achalasia“[Title/Abstract] OR ”Diffuse esophageal spasm“[Title/Abstract] OR ”Eosinophilic esophagitis“[Title/Abstract] OR ”Chagas disease“[Title/Abstract]) AND (“Phosphodiesterase 5 inhibitors“[Title/Abstract] OR ”PDE-5 inhibitors“[Title/Abstract] OR ”Sildenafil“[Title/Abstract] OR ”Tadalafil“[Title/Abstract] OR ”Vardenafil“[Title/Abstract]). Studies not identified by the above databases were included by evaluating the reference sections of relevant studies.

### Study selection and data extraction

We have included randomized clinical trials, observational studies (cross-sectional, case-control, or cohort), case series/reports, congress and conference abstracts as a source of grey literature. The following criteria were used as our inclusion criteria; (1) Studies included healthy subjects or patients with esophageal motility disorders; (2) Studies administered PDE-5 inhibitors (sildenafil, tadalafil, and vardenafil) as an intervention with or without a placebo group; (3) Studies reporting relevant outcomes reported from manometry including but not limited to LES pressure, contraction wave amplitude, and residual pressure. The title and abstract of the studies were assessed based on the inclusion criteria after duplicate papers were removed. Finally, a thorough screening of the full texts took place. The selection was carried out independently by the two authors (AS, RB). The same authors independently extracted the following data: author, year, country, type of study, population, number of total patients, intervention arm, control arm (if applicable), outcome, and adverse events. A third reviewer resolved disagreements (MT).

### Outcomes

Since the studies included evaluated the efficacy of PDE-5 inhibitors through assessing the values reported by manometry, three main outcomes were defined to be investigated in our study: (1) Lower esophageal sphincter (LES) pressure; (2) Amplitude of contractions produces by esophageal muscles; and (3) Residual pressure which assessed at the nadir of relaxation after each swallow. The results of other reported outcomes by manometry were narratively described in the results section.

### Quality assessment

The quality of the included studies was evaluated by using the checklist developed by the National Heart, Lung, and Blood Institute in 2013. The tool comprised of several checklists provided to assess a study’s internal validity. In our work, we used the checklists for interventional and before/ after studies [8]. In order to assess the quality of case reports, we used the checklists provided by JBI’s critical appraisal tool [9].

### Data synthesis

All data were retrieved as mean and standard deviations (SD). We then calculated our pooled effect sizes using standardized mean difference (SMD) with 95% confidence intervals (95% CI). Heterogeneity was evaluated by assessing I2 values and Cochrane Q test (with p value less than 0.1 showing heterogeneity). A random-effects meta-analysis was conducted because the methodology and the settings varied across studies. We performed a subgroup analysis dividing studies which evaluated the efficacy of PDE-5 inhibitors on healthy individuals and those evaluating on patients with esophageal motility disorders. A sensitivity analysis was performed to assess the individual effect of each study on the pooled effect size by omitting it from the pooled results (leave one out method). Publication bias was assessed for outcomes with at least 10 studies using funnel plot and egger’s regression test for plot asymmetry. All analyses were performed in R using meta package (version 5.5-0) [10].

## Results

**Fig. 1:**
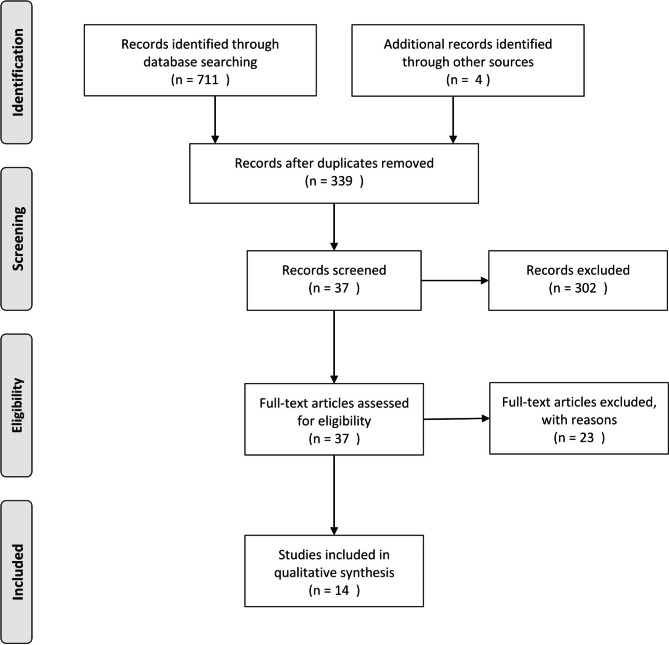
Evidence search and selection based on the PRISMA (Preferred Reporting Items for Systematic reviews and Meta-Analyses) approach

The systematic search yielded 711 studies through database searching and three studies through other sources. After removal of duplicate articles, examining titles and abstracts, and screening based on access to full texts, 14 studies were included and their data were extracted (Fig. 1). These articles assessed the effects of PDE-5 inhibitors on esophageal motility, mostly sildenafil [5, 6, 11–25].

### Characteristics of the included studies

The 14 included studies were conducted in seven different countries, with Korea (4 articles), Italy (3 articles), the United States (1 articles), Taiwan (2 articles), Austria (1 article), Brazil (1 article) and two studies with unknown origin (Table-1) [5, 6, 12–16, 18–22, 24, 25]. The included studies were published between 2000 and 2021. The study by Costa, T. (2020) was conducted in Brazil and had the largest sample size with 22 patients [15].

**Table 1:** Characteristics of included studies and quality assessment results

Author/Year	Country	Type of study	Population	Total Patients (n)	Age	Intervention arm(s)/N	Control arm/N	Outcome	Adverse events
Bortolotti, M/2000	Italy	RCT	Patients affected by achalasia with an esophageal diameter of < = 5 cm	14	Mean age, 41 years; age range, 21–64 years	A 50-mg tablet of sildenafil	Placebo	Sildenafil inhibits the contractile activity of the esophageal musculature of patients with achalasia, decreasing lower esophageal sphincter tone and residual pressure as well as contraction amplitude.	No side effects were observed, except for headache in one case.
Bortolotti, M/2001	Italy	RCT	Healthy subjects	16	34 years, age range 22–56	A 50-mg tablet of sildenafil	Placebo	Sildenafil markedly inhibits the motor activity of the esophageal musculature by decreasing LES pressure, wave amplitude, and propagation velocity	No side effects were observed, except for headache in one case.
Rhee, P.L./2001	Korea	Before/ After	Healthy male adult volunteers	8	Mean age = 30.5 years	A 50-mg tablet of sildenafil	N.A	LES resting pressure significantly decreased after sildenafil infusion. The body’s peristaltic amplitude gradually decreased and eventually disappeared	No serious side effects were noticed
Bortolotti, M. /2002	Italy	RCT	Patients with symptomatic hypertensive LES	14	Mean age 35 years, range 28–55	A 50-mg tablet of sildenafil	Placebo	Sildenafil inhibits the lower oesophageal sphincter tone and pressure wave amplitude of patients with symptomatic hypertensive LES	No side effects were observed
Eherer, A. J./2002	Austria	Before/ After	Healthy subjects, nutcracker esophagus, hypertensive LES, and achalasia patients	Healthy subjects: 6, Patients with esophageal disorder: 11	Normal subjects: aged 26–30 years, Patients: aged 27–57 years	A 50-mg tablet of sildenafil	Placebo	Sildenafil lowers LOS pressure and propulsive forces in the body of the esophagus of healthy subjects as well as in patients with nutcracker oesophagus, hypertensive LOS, and achalasia.	Two patients experienced side effects and did not want to continue treatment. (Side effects included: headaches, dizziness, fatigue, sleep disturbances, feeling of tightness in the chest at night.)
Mathis, C./2002	USA	Before/ After	Patients diagnosed with hypertensive LES	N.A	N.A	Sildenafil (50 mg) dissolved	N.A	Sildenafil inhibits LES tone and the amplitude of contractions of the distal esophageal body in patients with hypertensive LES.	N.A
Lee, J. I. / 2003	Korea	Before/ After	Healthy subjects, patients with nutcracker esophagus	Healthy subjects: 8, Patients with nutcracker esophagus: 9	Healthy subjects: 24.3 years, Patients with nutcracker esophagus: 44.7 years	0.8 mg/kg sildenafil dissolved in 20 mL of water	distilled water	In both healthy subjects and patients with nutcracker esophagus, sildenafil decreased resting LOS pressure and the amplitude of peristaltic pressure waves at 3, 8 and 13 cm above LOS. Sildenafil also prolonged the duration of LOS relaxation. It had no effect on the velocity of peristalsis or the amplitude of peristaltic pressure waves 18 cm above LOS.	No noticeable side-effects reported
Kim, H. S. / 2006	Korea	Before/ After	Healthy subjects	8	Mean age : 38.5 years	50-mg dose of sildenafil	N.A	Sildenafil decreased the resting lower esophageal sphincter pressure and prolonged the duration of lower esophageal sphincter relaxation for the 45 min following its ingestion, and does not induce gastro-oesophageal reflux.	N.A
Lee, T. H./ 2012	Korea	RCT	Healthy volunteers	16	Mean age : 31.4 years	10 mg vardenafil	Water	Vardenafil decreased resting and residual LES pressures, distal esophageal contraction, and bolus transit.	N.A
Rodriguez, R. / 2013	N.A	Before/ After	Healthy volunteers, patients with type II/III achalasia, and hypertensive peristalsis	20	Healthy group: mean age 27.5 Patients: mean age 45 years	Single dose of 20 mg tadalafil	N.A	Tadalafil is a 5-PDE that induce a prolonged and sustained effect on esophageal contractions, is well tolerated and it could be a promising drug in the management of esophageal motor disorders.	Mild headache
Guevara-Morales /2014	N/A	Before/ After	Achalasic Patients	12	N/A	20 mgs tadalafil	N/A	Tadalafil may significantly reduced LES pressure and IRP in achalasia patients, and patients with Type 2 Achalasia had a better response.	Headache and back pain
Costa, T./2020	Brazil	Before/ After	Healthy volunteers	22	Mean age : 38.1 years	50 mg sildenafil	N.A	Sildenafil caused a significant reduction in distal contractile integral and integrated relaxation pressure in the lower esophageal sphincter. In the proximal esophagus the alteration in distal esophageal contraction caused a significant increase in contraction length, contractile integral, and contraction duration; suggesting an adaptive compensation of the proximal esophagus for the effect of sildenafil on distal esophageal motility.	There was no adverse event after administration of sildenafil.
Wong, M. W. /2020	Taiwan	RCT	Healthy subjects	15	Mean age: 27 years	50 mg sildenafil	Placebo	Sildenafil attenuates esophagogastric junction barrier function, resting LES pressure, and LES relaxation. Both esophageal body contractility and contraction reserve are in- hibited by sildenafil in healthy adults.	Significant adverse effects were not observed in patients.
Wong, M. W. /2021	Taiwan	RCT	Healthy subjects	17	Mean age : 30.2 years	50 mg sildenafil	Placebo	Sildenafil reduces both the success rate and the vigor of secondary peristalsis, similar to that seen with primary peristalsis.	N.A

### Qualitative synthesis

In the 14 included studies, two trials comprised healthy control subjects alongside patients with esophageal motility disorders [13, 14], five studies conducted as a before/after trial on patients with esophageal motility disorders [16, 18, 20, 22, 25], and four trials conducted as RCT on healthy subjects [5, 6, 12, 21]. Sildenafil was used in 11 studies [5, 6, 12–16, 19, 20, 22, 24]; one study only used vardenafil [21], and two studies only used tadalafil [18, 25].

### Quantitative synthesis

#### Lower esophageal sphincter (LES) pressure

**Fig. 2:**
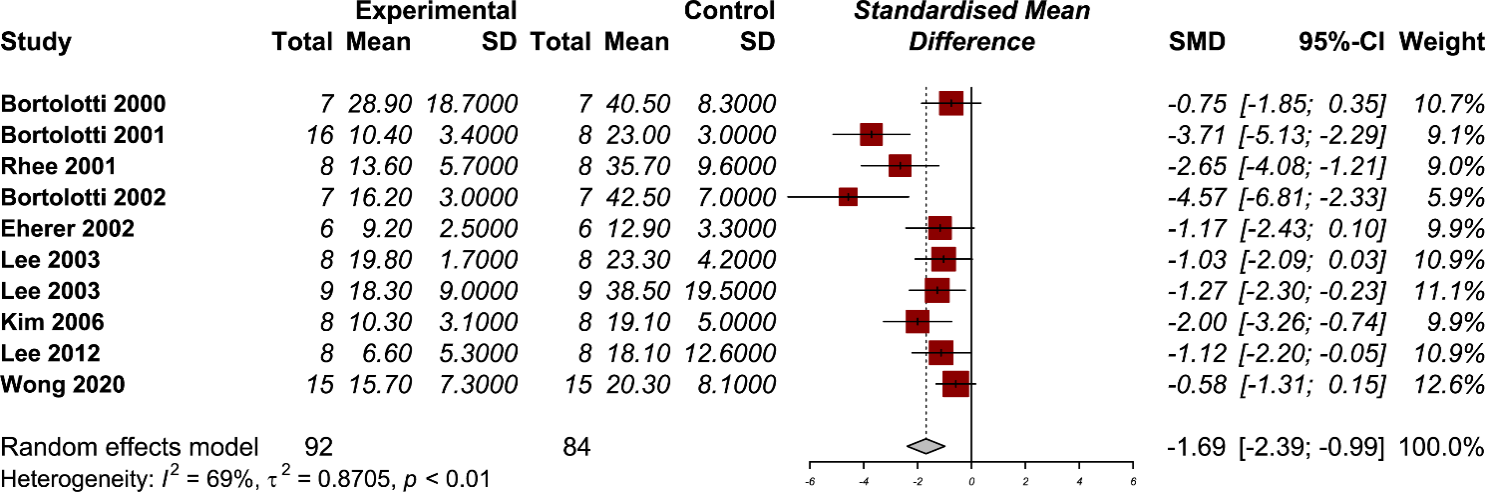
Forest plot for the efficacy of phosphodiesterase 5 (PDE-5) inhibitors on lower esophageal sphincter pressure

Nine studies have reported the LES pressure after using PDE-5 inhibitors in their manometry results [5, 12–14, 16, 19–21, 24]. The use of PDE-5 inhibitors was associated with a statistically significant reduction in the LES pressure (SMD − 1.69, 95% CI: -2.39 to -0.99) (Fig. 2). The heterogeneity between studies were significant (P < 0.01) with moderate amount of heterogeneity (I2: 69%). Our subgroup analysis dividing studies that were conducted on healthy individuals and those who had esophageal motility disorders showed a non-significant between group differences (P = 0.77) (Supplementary material). The results of our sensitivity analysis showed no significant changes in the pooled effect size after omitting each study from the overall analysis (Supplementary material). A funnel plot asymmetry was detected by Egger’s test (P = 0.001, Supplementary material), showing the findings may be subjected to publication bias.

### Amplitude of contractions

**Fig. 3:**
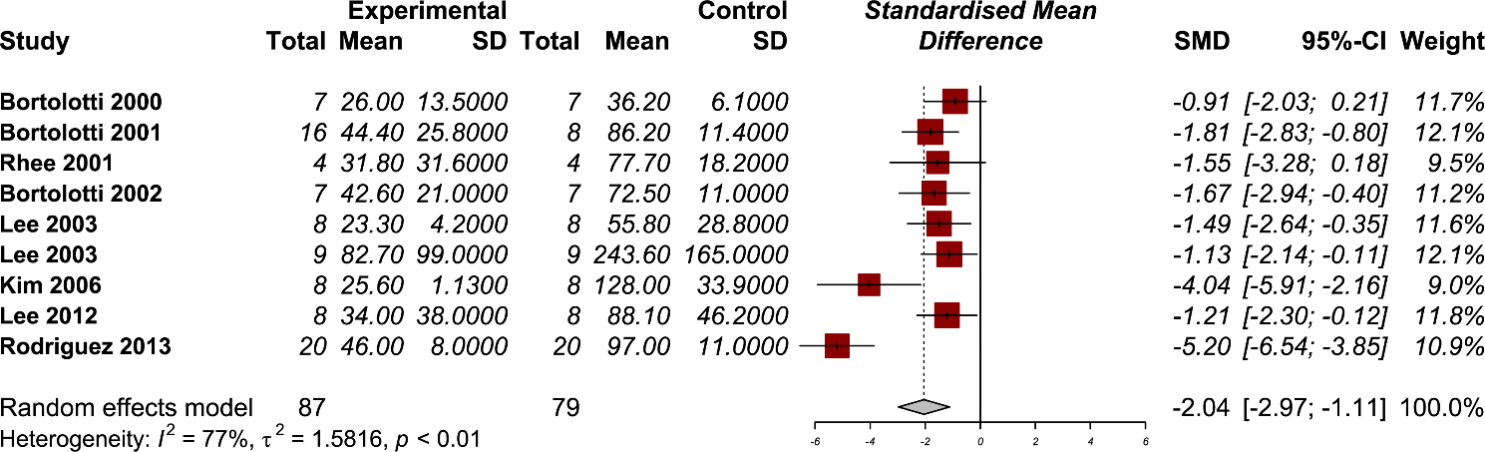
Forest plot for the efficacy of phosphodiesterase 5 (PDE-5) inhibitors on the amplitude of contractions

Eight studies have reported the amplitude of contractions after using PDE-5 inhibitors in their manometry results [12–14, 19–21, 24, 25]. The use of PDE-5 inhibitors was associated with a statistically significant reduction in the amplitude of contractions (SMD − 2.04, 95% CI: -2.97 to -1.11) (Fig. 3). The heterogeneity between studies were significant (P < 0.01) with considerable amount of heterogeneity (I2: 77%). The results of subgroup analysis showed that using PDE-5 inhibitors in reducing the amplitude of esophageal contractions were significantly lower in healthy individuals (SMD − 2.50, 95% CI: -3.81 to -1.18) compared with those with esophageal motility disorders (SMD − 1.19, 95% CI: -1.84 to -0.55) (Supplementary material). The results of our sensitivity analysis showed no significant changes in the pooled effect size after omitting each study from the overall analysis (Supplementary material).

### Residual pressure

**Fig. 4:**
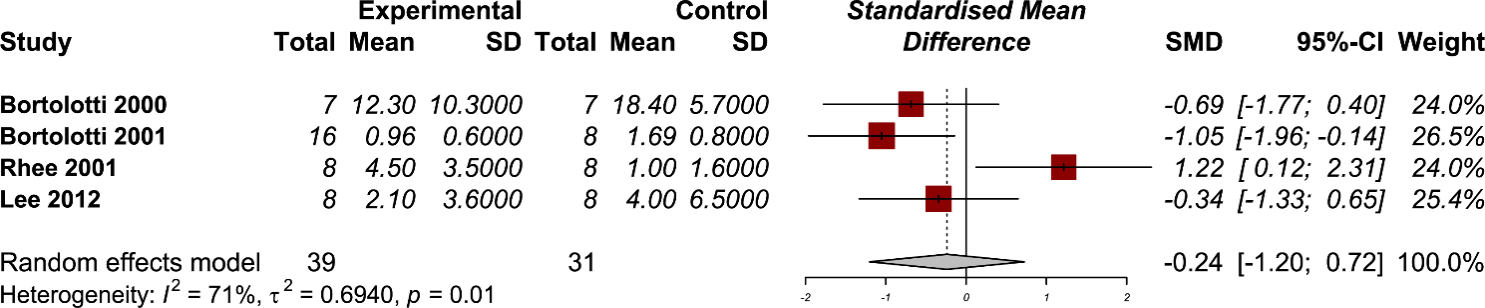
Forest plot for the efficacy of phosphodiesterase 5 (PDE-5) inhibitors on residual pressure

Four studies have reported the residual pressure of esophagus after using PDE-5 inhibitors in their manometry results [12, 13, 21, 24]. There was no significant effect of PDE-5 inhibitors in reducing the residual pressure (SMD − 0.24, 95% CI: -1.20 to 0.72) (Fig. 4). The heterogeneity between studies were significant (P = 0.01) with moderate amount of heterogeneity (I2: 71%). The results of subgroup analysis showed no significant between group differences (P = 0.49) (Supplementary material). The results of our sensitivity analysis showed a significant change in the pooled effect size after omitting the study by Rhee et al. [24] from the overall analysis (Supplementary material).

### Adverse events

Overall, the reports regarding the side effects regarding the use of PDE-5 inhibitors in patients with esophageal motility and healthy patients shows that using these drugs is accompanied with no serious adverse effects. Most reported side effect among patients were headache. Only two patients in the study by Ehrer et al. decided to discontinue the usage because of side effects they were experiencing [16].

### Quality assessments

The results of our quality assessment showed that among the included reports, there were no study with poor methodology. There were 4 and 7 studies with fair and good quality, respectively. The quality of three conference abstracts were not evaluated due to limited information about their methodology [18, 22, 25]. The most important limitation found were small sample sizes assessed in trials. Absence of healthy control group to compare the results with those who had esophageal disorders and conducting trials only on healthy individuals were other important limitation of the included studies. The detailed results of the quality assessment are available in supplementary material Table 1.

## Discussion

The articles included in this systematic review represent that the PDE5 inhibitors including sildenafil, vardenafil or tadalafil result into manometric alterations such as significantly decreased LES tone, wave amplitude and residual pressure of the esophagus. From our analyses, the PDE5 inhibitors are safe and effective medications as a treatment option for esophageal motility disorders. Of note, serious adverse effects of PDE5 inhibitors were not seen in the patients although some patients experienced fatigue, dizziness, headaches, feeling of tightness in the chest or sleep disturbance. There were various ways for implementation of PDE5 inhibitors to patients including 25 mg or 50 mg tablets of sildenafil, dissolved sildenafil, 0.8 mg/kg sildenafil dissolved in 20ml of water, 10 mg vardenafil and 10 mg or 20 mg tablets of tadalafil.

To the best of our knowledge, this is the first study reviewing and representing evidence for PDE-5 inhibitors efficacy in esophageal motility disorders including achalasia, hypertensive LES, and nutcracker esophagus. PDE5 inhibitors inhibit the contraction of smooth muscles by maintaining the accumulation of cGMP, which is generated by NO. The known therapeutic effect of PDE5 inhibitors is in the treatment of erectile dysfunction, where they inhibit the human corpus cavernosum arterioles smooth muscle cells [26]. It is evident that the concentration of cGMP is pivotal in the motor function control of the esophagus. The changes in the motility of distal esophagus are associated with changes in the proximal esophagus for solid and liquid bolus. It has been reported that PDE-5 inhibitors such as sildenafil inhibit esophageal propulsive forces, decrease LES pressure, and delay the gastric emptying rate [27, 28]. There is insufficient evidence regarding one preferred treatment option for esophageal motility disorders over the others, indicating that the concern for the best treatment option for esophageal motility disorders remains challenging. Alongside PDE5 inhibitors, other medical treatments are being used, including nitrates, sedatives, anticholinergics, tricyclic antidepressants, calcium channel blockers, and serotonin-reuptake inhibitors[17, 29–33]. The complications and adverse events of administration of PDE-5 inhibitors are not well discussed in detail in other publications. However, in the study by Kim et al. [34], 300 patients were given sildenafil for the treatment of erectile dysfunction. The most reported adverse effects were headache and facial flushing that lasted less than one hour. In another study by Galie and colleagues [35], the side effects of sildenafil in pulmonary artery patients were headache, flushing, and dyspepsia in 45%, 11.6%, and 11.6% of the patients, respectively. Also, it was reported that there was not a correlation between the dosage of sildenafil and tolerability of the medication.

During our database search, we have found 3 case reports on this topic [11, 17, 23]. In a case report by Agrawal A. et al.[11] a 37-yer-old man with mixed motility abnormality diagnosed by esophageal manometry was included. His complain was four or five severe retrosternal chest pain episodes per day for three years. In the first step, 50 mg of sildenafil was given to the patient and he reported that his chest pain episodes decreased to one mild chest pain episode per day. The treatment was repeated with 10 mg of vardenafil and also 10 mg of tadalafil with at least one-week interval between the change in the medication. The similar rapid and dramatic response to treatment was seen with the use of vardenafil or tadalafil. In the case report by Miller et al.[23], the patient was a 55-year-old woman with severe corkscrew esophagus who had dysphagia with liquids and solids for 3 years and progressive worsening for 6 months. The treatment was prescribing 25 mg orally administered tablets of sildenafil, twice per day. The patient then reported the resolution of her dysphagia and chest pain for the first time after the initial symptoms of her disease. In addition, the patient had continued symptoms control with no acute complaints. The manometry reports from two patients studied by Fox and colleagues showed using 25–50 mg BID sildenafil in patients with esophageal spasm could ameliorate focal and diffused spasm which is the primary cause of their symptoms (which was dysphagia and chest pain) without any significant adverse effects. The symptoms were resolved during the period that patients were taking the drug [17].

It is worth mentioning the included studies in this review assessed the esophageal effect of sildenafil among both patients with disorder and those who were healthy. Combining data from these articles may impose the results of our meta-analysis to bias. Therefore, it is important to take this issue in consideration. A recent study by Wong et al.[6] reported that the success rate of complete primary peristalsis was 100% after the use of sildenafil in all participants. The participants were 17 healthy volunteers including 15 men and 2 women who received either 50 mg of sildenafil or a placebo on two separate days with an interval of at least one week. In the same study, the secondary peristalsis’s success rate after using sildenafil was significantly lower than with the placebo. The pressure wave amplitude in the study by Rhee et al.[24] was significantly lower in the proximal and also distal esophagus after the sildenafil infusion. The procedure was done on eight healthy male adults with no history of using medications, smoking, or cardiovascular diseases. Basal and follow-up manometry was done after the infusion of sildenafil. Lee et al. [20] reported a decrease in amplitude of peristaltic pressure waves only at 3.8 to 13 cm above the LES. One study [13] reported the residual pressure of the esophagus after the infusion of sildenafil. Fourteen patients including eight men and six women with evidence of idiopathic achalasia were enrolled in the study. A tablet of 50 mg sildenafil ground and dissolved in 10 mL water or a placebo was given to the patients forming two groups of seven. The LES residual pressure values were not significantly different between the sildenafil and placebo groups at the post-infusion period. In the Brazilian study [15] it was demonstrated a significant increase in proximal and also distal contractile integral after sildenafil intake. Contractile integral was defined as the amplitude of contraction * duration of contraction * length of contraction. In this study, the ingestion of sildenafil lead to a significant reduction in distal contractile integral and a significant increase in proximal contractile integral. The post-infusion propagation velocity of pressure waves was significantly lower than the basal period in the results of one study[12]. By dividing the distance between the most proximal and distal recording ports of the esophagus by the interval between the beginning of the respective pressure waves, propagation velocity was determined. The study was performed on healthy subjects including nine men and seven women. All subjects experienced an overnight fasting. Then a placebo tablet was given to eight subjects forming placebo group and a tablet of 50 mg sildenafil dissolved in 10 cc of water was infused in the stomach of eight subjects forming sildenafil group. The propagation velocity of pressure waves was significantly lower after the use of sildenafil than the basal period between the sildenafil and placebo group but had not a significant difference between two groups. A study by Eherer AJ et al.[16] suggests that sildenafil reduces the primary peristalsis of the esophagus using conventional manometry. Secondary peristalsis is often triggered by local distention stimulation leading to an afferent signal of the swallowing center by the vagus nerve. The motor consequence between the primary and secondary peristalsis is similar while the control mechanisms or pathways are different. It is demonstrated that the vigor of the primary peristalsis is higher than the secondary peristalsis[36–38]. In the same study by Eherer AJ et al.[16] it has been reported that sildenafil reduces the primary peristalsis amplitude measuring with conventional manometry[5]. Sildenafil also inhibits the contraction reserve and body contractility of the esophagus compared with placebo[5]. The analyses revealed that sildenafil has no inhibitory effect on the proximal esophagus or the upper esophageal sphincter.

Most of the evidence available from PDE-5 inhibitors are limited to sildenafil. Only 4 studies evaluated other PDE-5 inhibitors except sildenafil [11, 18, 21, 25]. In the study by Rodriguez R. et al.[25] 10 healthy volunteers and 10 patients with type II or III achalasia or hypertensive peristalsis were included and received 20 mg of tadalafil. In the control group, the esophageal pressure reduction was observed in nine subjects and in eight patients in the patient group. Six patients declared improvement of dysphagia at 24 and 48 h and three patients reported this improvement at 72 h after the use of tadalafil. In all patients, tadalafil had no cardiovascular effect. Guevara-Morales et al. [18] aimed to investigate the possible effect of tadalafil administration on the LES basal pressure and integrated relaxation pressure (IRP) of 12 patients with achalasia. Six patients with type 2, three patients with type 1, and three patients with type 3 achalasia were evaluated every two weeks for two months. The before-after results of their study revealed a significant effect of tadalafil on LES and IRP. Moreover, the majority of patients did not have any signs of dysphagia after they were treated. In the vardenafil study by Lee et al.[21] 16 healthy volunteers divided into distilled water or vardenafil group. The resting and residual pressure of LES were significantly differed only in the vardenafil group.

There have been few studies evaluating the effect of PDE5 inhibitors other than sildenafil, including vardenafil of tadalafil. Four out of the seventeen included studies examined the effect of vardenafil or tadalafil with the same result as sildenafil. Previous studies have demonstrated that sildenafil causes a significant increase in the latency for peristalsis of the esophagus[39, 40]. On the other hand, vardenafil significantly decreases the resting and residual LES pressure and also distal esophageal contraction. Vardenafil also decreases the esophageal bolus transit when in a seated position despite decreased LES pressure[21]. The effects of tadalafil last up to 36 h although it has the same course of action suggesting that it might provide the best coverage in the treatment of patients with hypercontractile esophageal motility disorders.

### Limitations

There are several reasons regarding the heterogeneity and also conflicting results among the included studies. First, all of the reviewed studies had relatively small sample sizes and further evaluation with large scale and well-designed studies should be conducted regarding the efficacy of PDEI-5s. Second, most of the reviewed studies were using only sildenafil and more trials are needed to evaluate the efficacy of other PDEI-5s including vardenafil or tadalafil leading to a precise conclusion. Third, the pooled results of our meta-analysis included investigated the effect of PDE-5 inhibitors on the esophageal motility of both healthy individuals and patients with esophageal motility disorder. Therefore, the findings must be interpreted with caution and future studies, specifically among patients with esophageal motility disorder, are needed on this topic.

## Conclusion

In this study, we have reviewed 14 studies that directly addressed the issue of effectiveness of sildenafil, vardenafil or tadalafil on esophageal peristaltic vigor and their effects on LES resting pressure. Current evidence from studies shows phosphodiesterase-5 inhibitors significantly reduce LES resting pressure and esophageal peristaltic vigor, meaning that esophageal body contractility and contraction reserve are inhibited by PDEI-5s .

## Electronic supplementary material

Below is the link to the electronic supplementary material.

Supplementary Material 1

## Data Availability

Data sharing is available by contacting corresponding author.
