# Supplementary material for: Phosphodiesterase 5 (PDE-5) inhibitors (sildenafil, tadalafil, and vardenafil) effects on esophageal motility: a systematic review
Source: BMC Gastroenterol. 2023 May 22;23:170. doi: 10.1186/s12876-023-02787-3 (PMC10201782; doi:10.1186/s12876-023-02787-3)

# Supplementary Material

**Phosphodiesterase 5 (PDE-5) inhibitors (sildenafil, tadalafil, and vardenafil) effects on esophageal motility: a scoping review**

**Supplementary Table 1.** Results of quality assessments.

**Supplementary Figure 1.** Forest plot for the results of the subgroup analysis for the efficacy of PDE-5 inhibitors on lower esophageal sphincter pressure.

**Supplementary Figure 2.** Results of the sensitivity analysis for the efficacy of PDE-5 inhibitors on lower esophageal sphincter pressure.

**Supplementary Figure 3.** Funnel plot for the efficacy of PDE-5 inhibitors on lower esophageal sphincter pressure.

**Supplementary Figure 4.** Forest plot for the results of the subgroup analysis for the efficacy of PDE-5 inhibitors on the amplitude of contractions.

**Supplementary Figure 5.** Results of the sensitivity analysis for the efficacy of PDE-5 inhibitors on the amplitude of contractions.

**Supplementary Figure 6.** Forest plot for the results of the subgroup analysis for the efficacy of PDE-5 inhibitors on the residual pressure.

**Supplementary Figure 7.** Results of the sensitivity analysis for the efficacy of PDE-5 inhibitors on the residual pressure.

This supplemental material has been provided by the authors to give readers additional information about their work.

**Supplementary Table 1.** Results of quality assessments.

| Trial | 1. Randomized | 2. Method of randomization | 3. Treatment allocation concealed | 4. Study participants and providers blinded | 5. People assessing the outcomes blinded | 6. Groups similar at baseline | 7. Overall drop-out rate from the study at endpoint 20% or lower | 8. Differential drop-out rate (between treatment groups) at endpoint 15 percentage points or lower | 9. High adherence to the intervention | 10. Other interventions avoided | 11. Outcomes assessed using valid and reliable measures | 12. Power description | 13. Outcomes reported or subgroups analyzed prespecified | 14. Intention-to-treat analysis | Overall |
| --- | --- | --- | --- | --- | --- | --- | --- | --- | --- | --- | --- | --- | --- | --- | --- |
| Bortolotti 2000 | Yes | No | CD | Yes | Yes | Yes | Yes | Yes | Yes | Yes | Yes | No | No | CD | Good |
| Bortolotti 2001 | Yes | No | CD | Yes | Yes | Yes | Yes | Yes | Yes | Yes | Yes | No | No | CD | Good |
| Bortolotti 2002 | Yes | No | CD | Yes | Yes | Yes | Yes | Yes | Yes | Yes | Yes | No | No | CD | Good |
| Lee 2012 | Yes | No | CD | Yes | Yes | Yes | Yes | Yes | Yes | Yes | Yes | No | No | CD | Good |
| Wong 2020 | Yes | No | CD | Yes | Yes | Yes | Yes | Yes | Yes | Yes | Yes | Yes | No | CD | Good |
| Wong 2021 | Yes | No | CD | No | No | Yes | Yes | Yes | Yes | Yes | Yes | Yes | No | CD | Fair |

**Supplementary Table 1.- Continued** Results of quality assessments.

| Before/ After | 1. Study question or objective clear | 2. Eligibility/selection criteria for the study population prespecified | 3. Representative | 4. All eligible participants enrolled | 5. Sample size sufficiently large | 6. Test/service/intervention clearly described | 7. Outcome measures prespecified, clearly defined, valid, reliable, and assessed consistently | 8. People assessing the outcomes blinded | 9. Loss to follow-up after baseline 20% or less | 10. Statistical methods examine changes | 11. Outcome measures of interest taken multiple times | 12. Individual-level data to determine | Overall |
| --- | --- | --- | --- | --- | --- | --- | --- | --- | --- | --- | --- | --- | --- |
| Rhee 2001 | Yes | Yes | No | Yes | No | Yes | Yes | No | Yes | Yes | No | NA | Fair |
| Eherer 2002 | Yes | Yes | Yes | Yes | No | Yes | Yes | Yes | Yes | Yes | No | NA | Good |
| Lee 2003 | Yes | Yes | Yes | Yes | No | Yes | Yes | Yes | Yes | Yes | Yes | NA | Good |
| Kim 2006 | Yes | Yes | No | Yes | No | Yes | Yes | No | Yes | Yes | Yes | NA | Fair |
| Costa 2020 | Yes | Yes | No | Yes | No | Yes | Yes | No | Yes | Yes | No | NA | Fair |

**Supplementary Figure 1.** Forest plot for the results of the subgroup analysis for the efficacy of PDE-5 inhibitors on lower esophageal sphincter pressure.


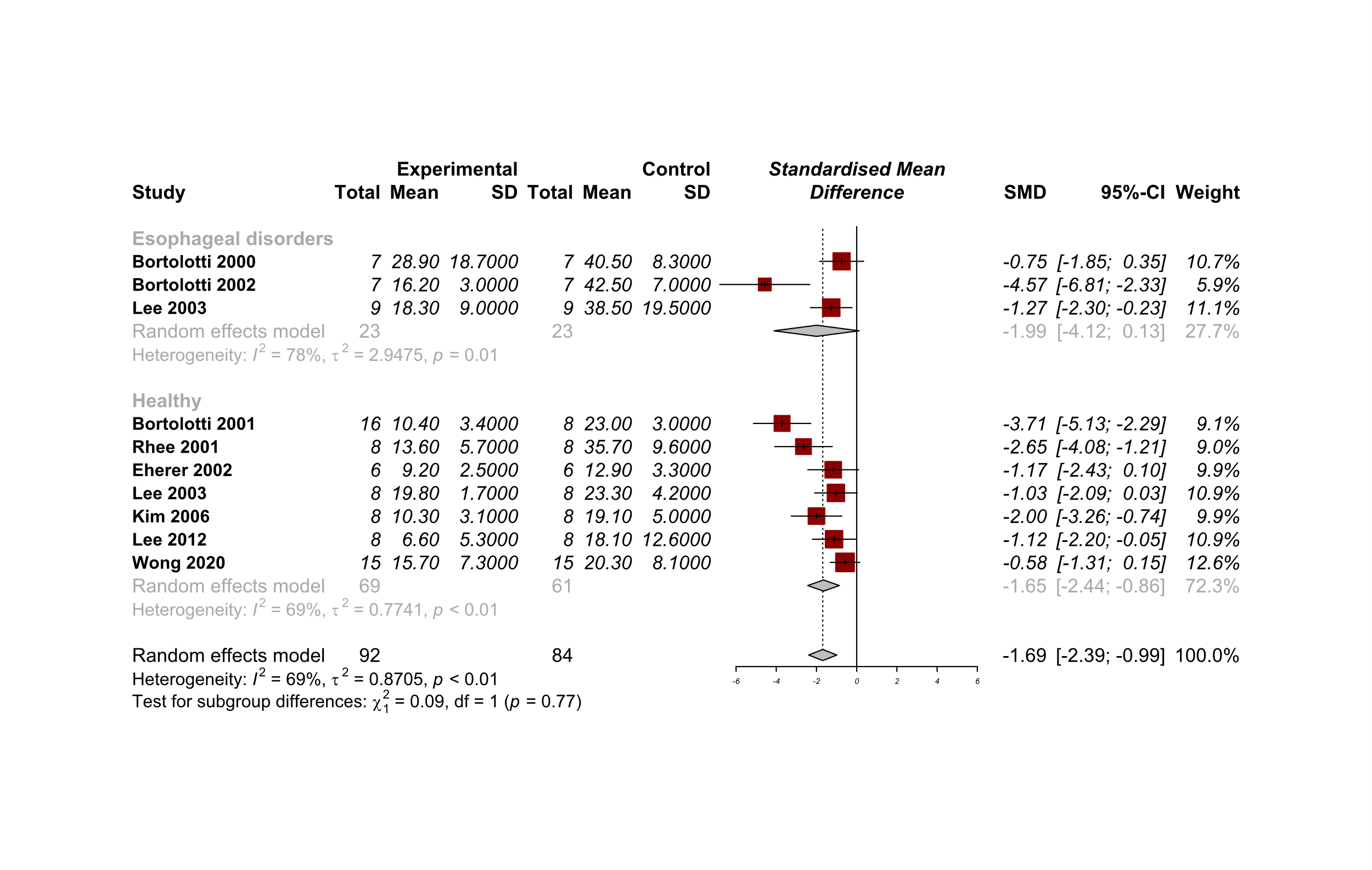


**Supplementary Figure 2.** Results of the sensitivity analysis for the efficacy of PDE-5 inhibitors on lower esophageal sphincter pressure.


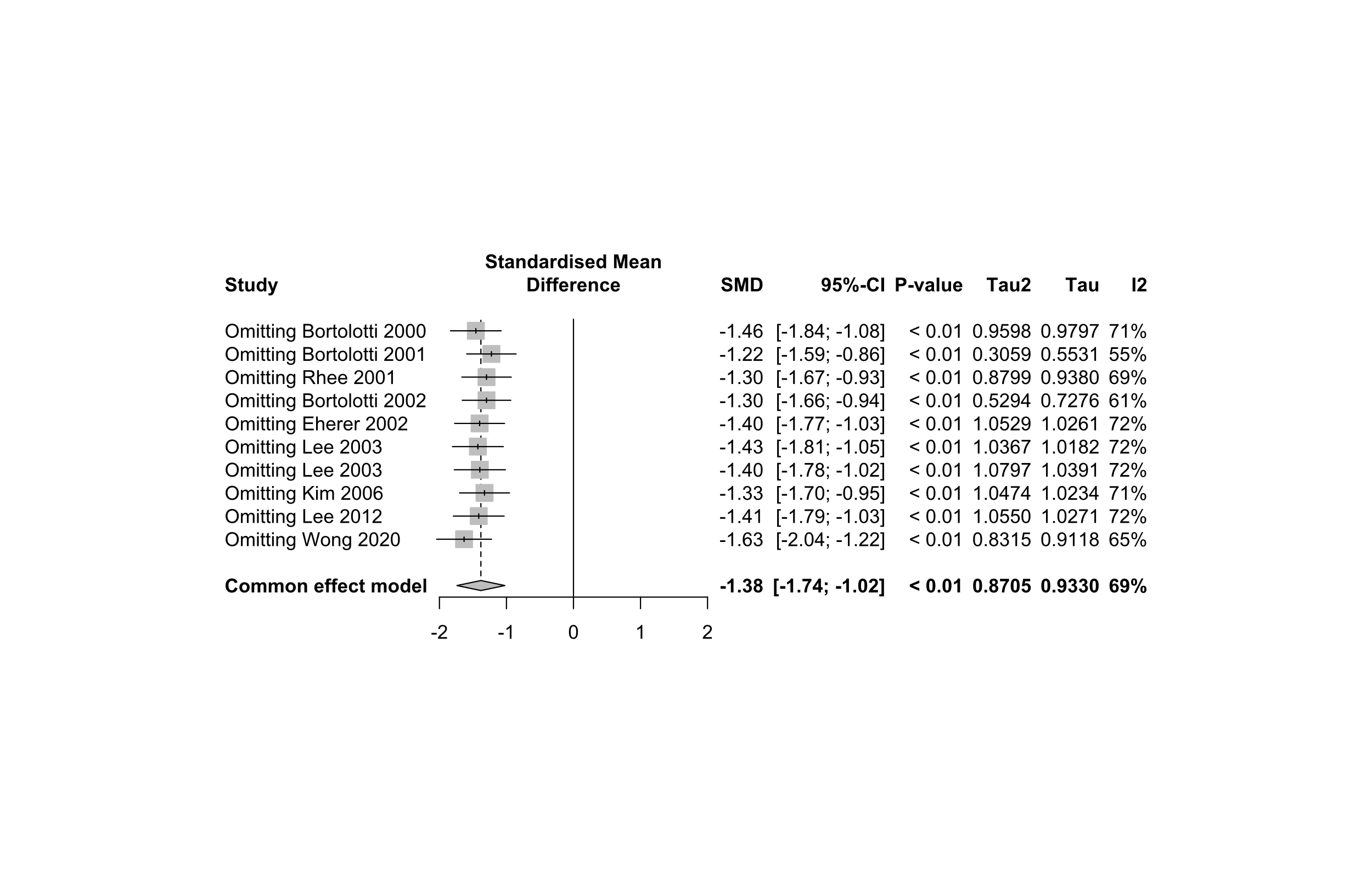


**Supplementary Figure 3.** Funnel plot for the efficacy of PDE-5 inhibitors on lower esophageal sphincter pressure.


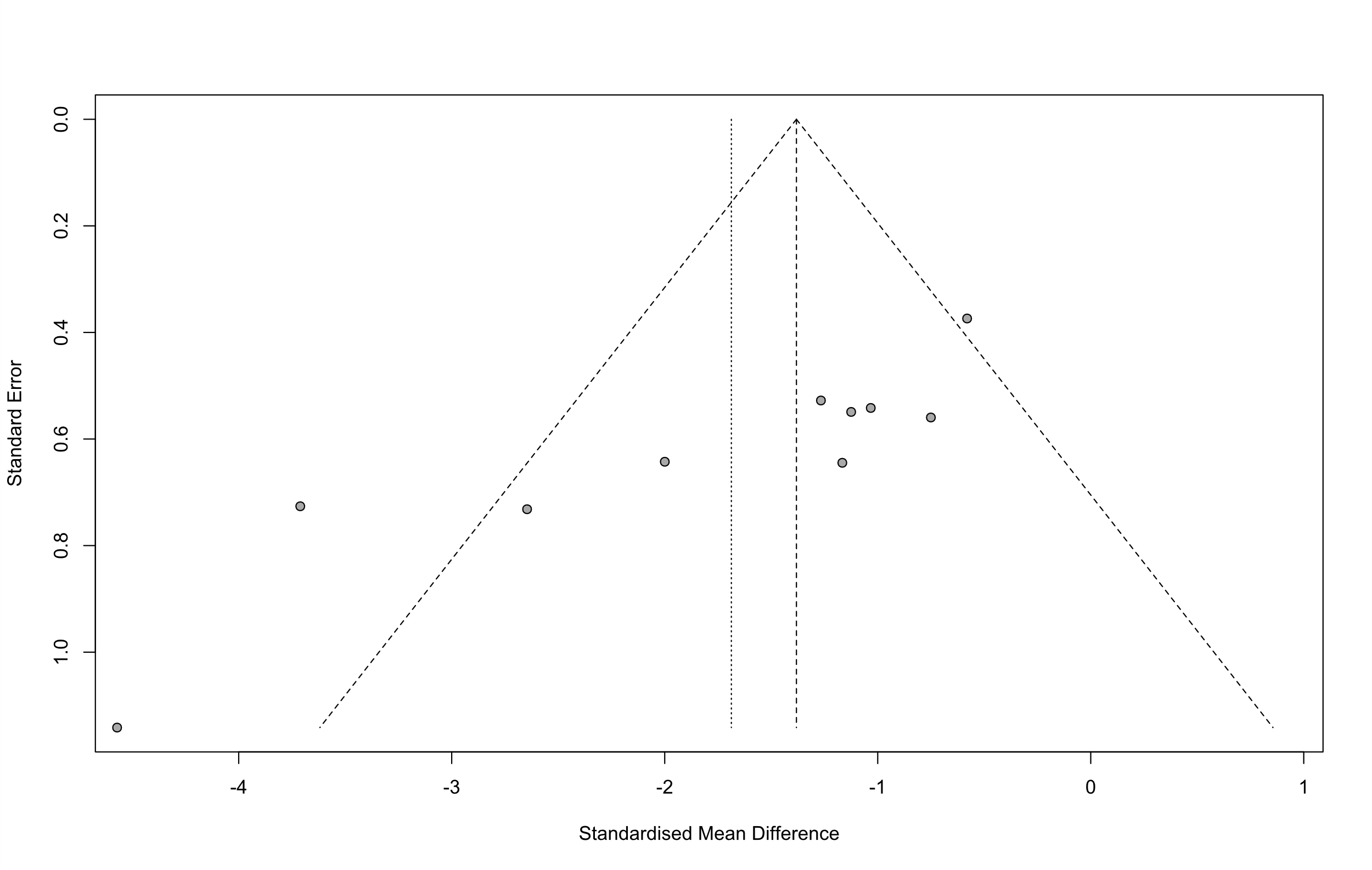


**Supplementary Figure 4.** Forest plot for the results of the subgroup analysis for the efficacy of PDE-5 inhibitors on the amplitude of contractions.


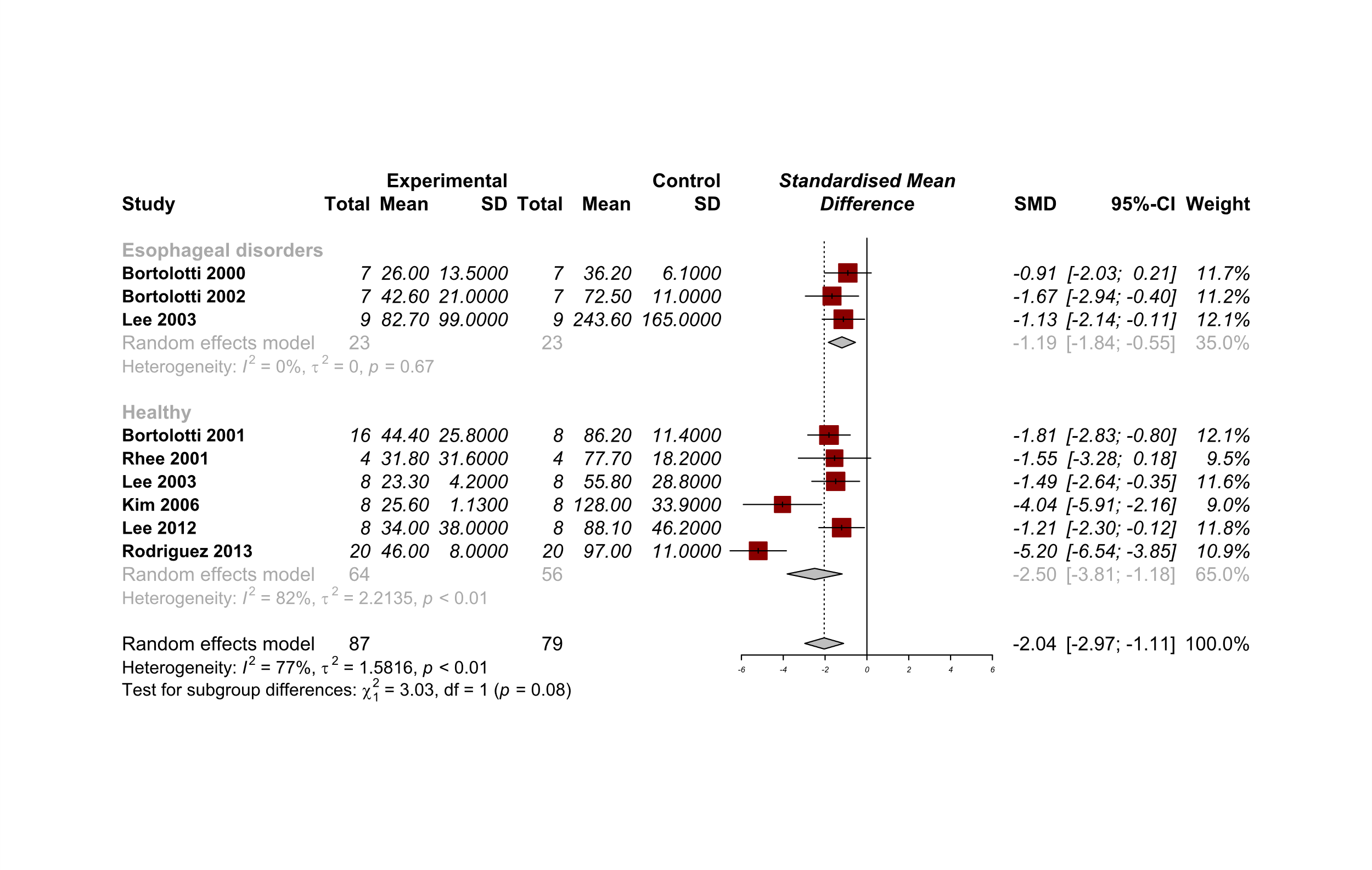


**Supplementary Figure 5.** Results of the sensitivity analysis for the efficacy of PDE-5 inhibitors on the amplitude of contractions.


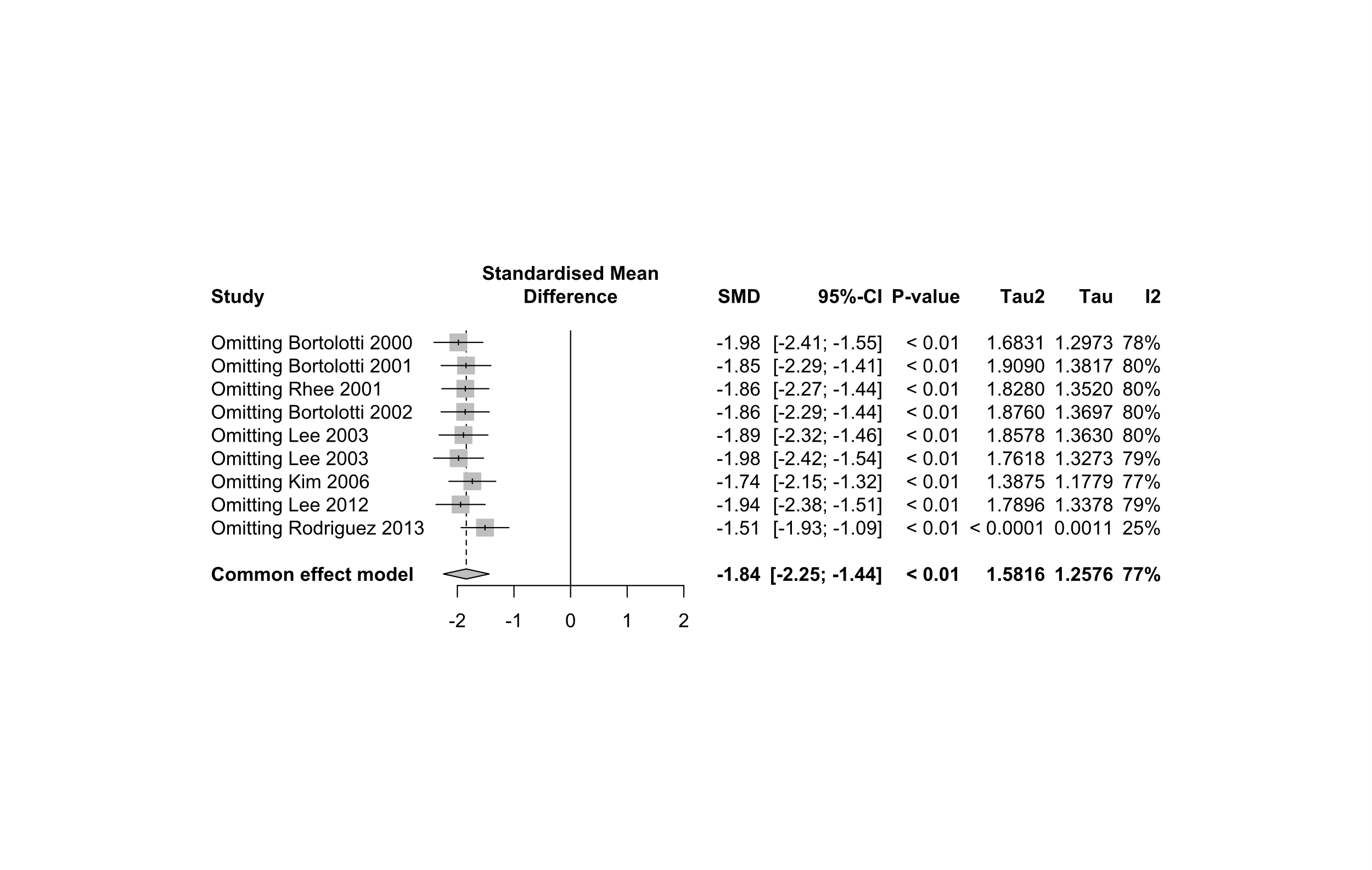


**Supplementary Figure 6.** Forest plot for the results of the subgroup analysis for the efficacy of PDE-5 inhibitors on the residual pressure.


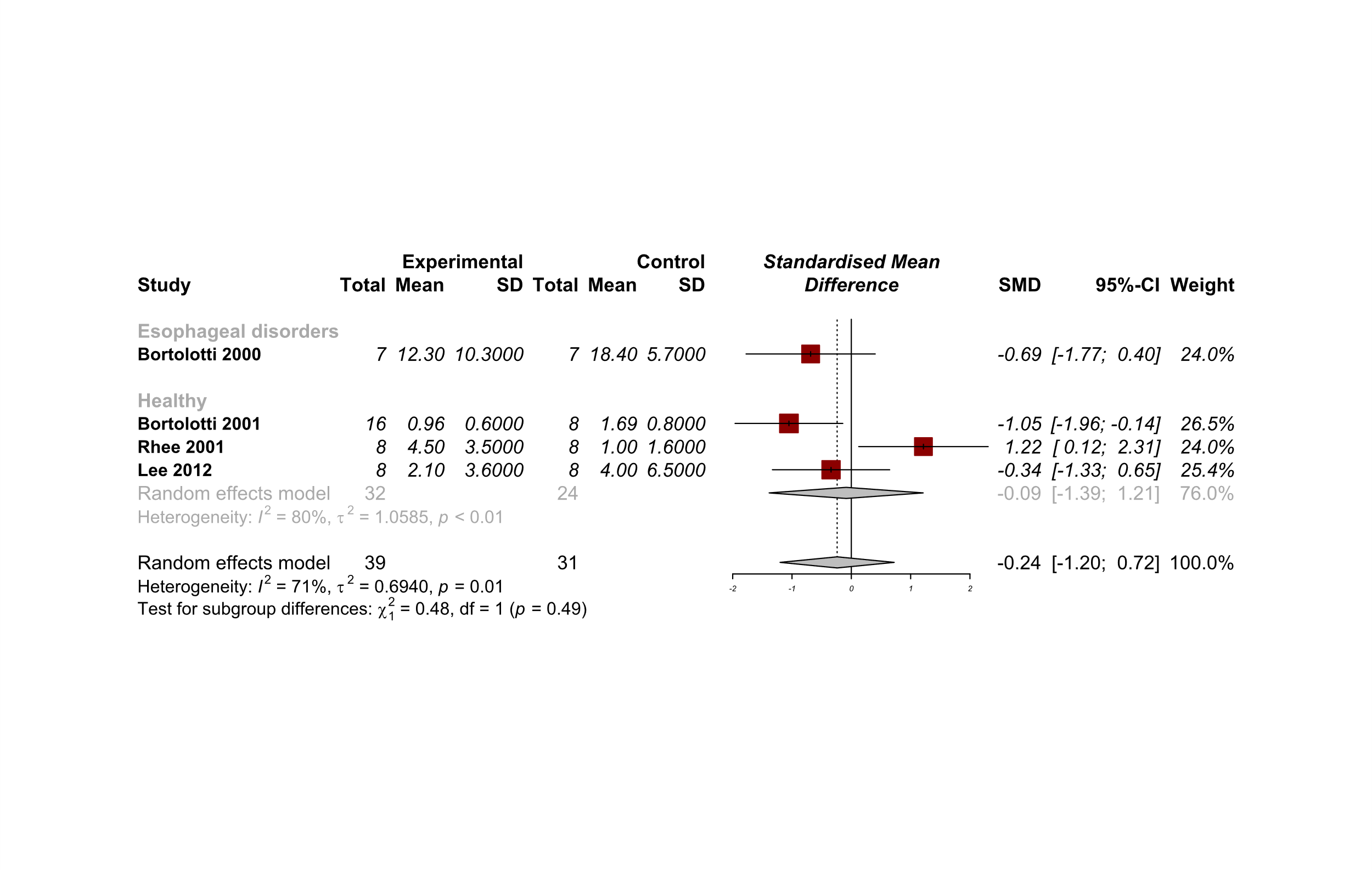


**Supplementary Figure 7.** Results of the sensitivity analysis for the efficacy of PDE-5 inhibitors on the residual pressure.


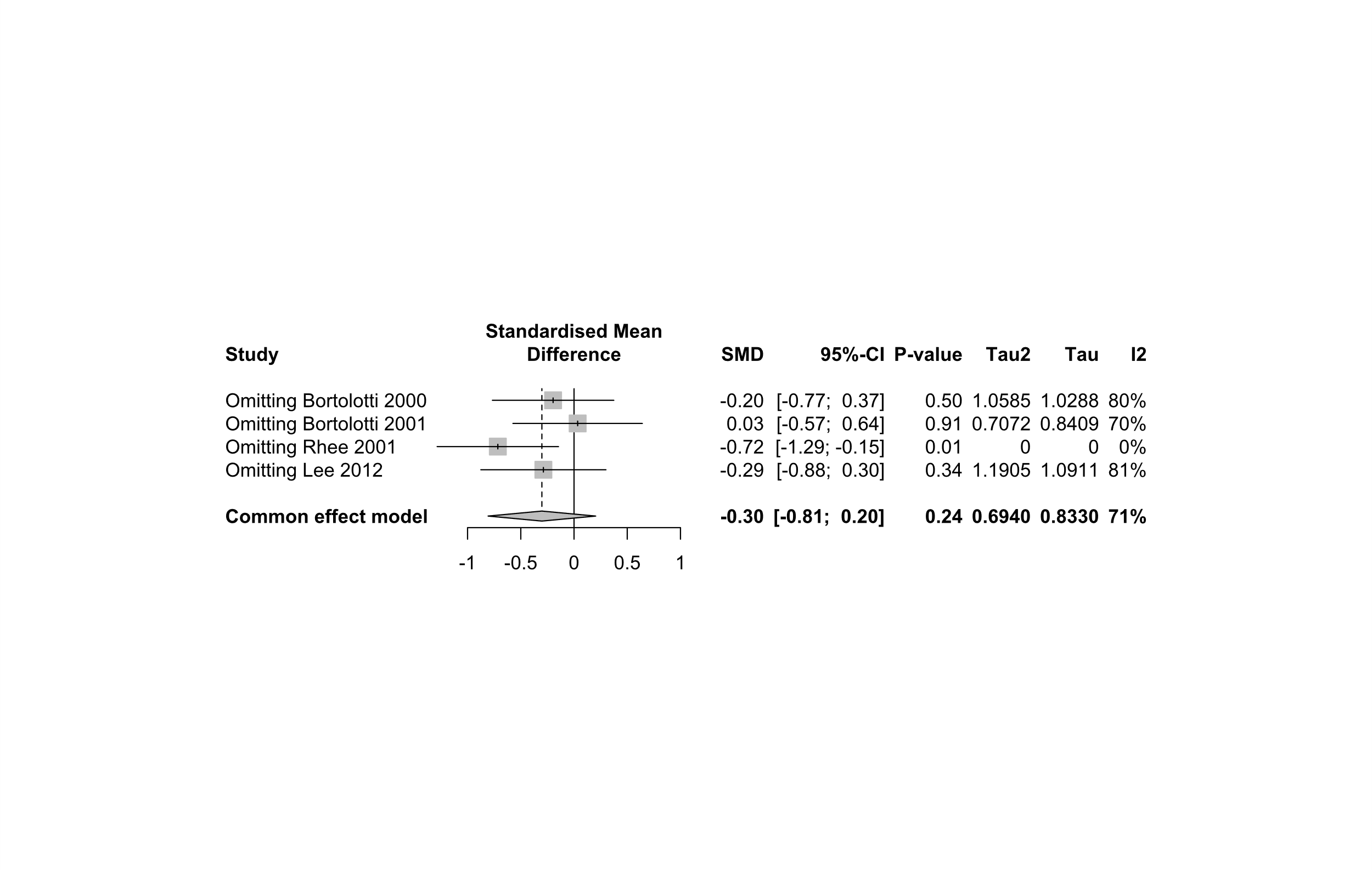

Supplement: Supplementary file 1 — Supplementary Material 1 [file 12876_2023_2787_MOESM1_ESM.docx]
